# P-2066. Quality of Life and Sociodemographic Characteristics of HIV-Infected Adults Receiving Antiretroviral in Venezuela: preliminary data

**DOI:** 10.1093/ofid/ofaf695.2230

**Published:** 2026-01-11

**Authors:** Gustavo Fernandez, Grecia Erimee, Cristina Estrabao, Yessica Goncalves, Isabella Garancini, Leslie Azaf, Gabriel Gomez, Oriana Gutierrez, Rohit Sharma, Andrés F Henao Martínez, David Forero-Peña

**Affiliations:** Universidad Central de Venezuela "Escuela de Medicina Luis Razetti", Caracas, Distrito Federal, Venezuela; Universidad Central de Venezuela "Escuela de Medicina Luis Razetti", Caracas, Distrito Federal, Venezuela; Universidad Central de Venezuela "Escuela de Medicina Luis Razetti", Caracas, Distrito Federal, Venezuela; Universidad Central de Venezuela "Escuela de Medicina Luis Razetti", Caracas, Distrito Federal, Venezuela; Universidad Central de Venezuela "Escuela de Medicina Luis Razetti", Caracas, Distrito Federal, Venezuela; Universidad Central de Venezuela "Escuela de Medicina Luis Razetti", Caracas, Distrito Federal, Venezuela; Universidad Central de Venezuela "Escuela de Medicina Luis Razetti", Caracas, Distrito Federal, Venezuela; Departamento de Medicina Familiar y Comunitaria, Hospital de Xátiva, Valencia, España., Valencia, Comunidad Valenciana, Spain; Mass General Brigham, Boston, Massachusetts, US, Boston, Massachusetts; University of Colorado Anschutz Medical Campus, Aurora, Colorado; Biomedical Research and Therapeutic Vaccines Institute, Ciudad Bolívar, Venezuela, Caracas, Distrito Federal, Venezuela

## Abstract

**Background:**

HIV is now a manageable chronic illness with near-normal life expectancy when Antiretroviral Therapy (ART) is taken correctly. However, social challenges such as poverty, stigma, and mental health issues continue to impact ART adherence and Quality of Life (QoL). In Venezuela, research in this area is scarce, with only one QoL study in the past decade. We assessed QoL using the EuroQol 5 Dimension (EQ-5D) scale and compared ART-adherent vs. non-adherent patients.

**Figure:**
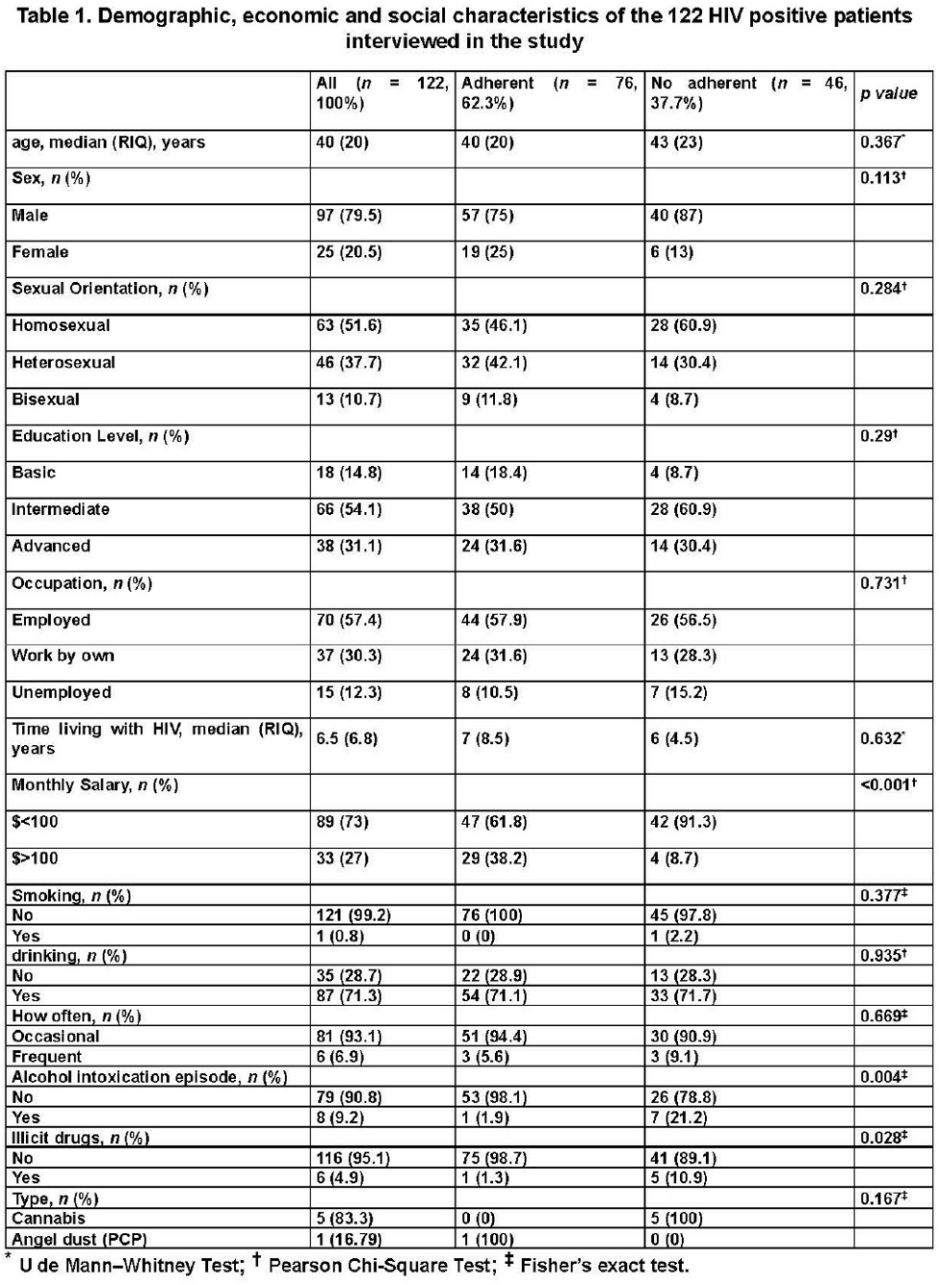

**Figure:**
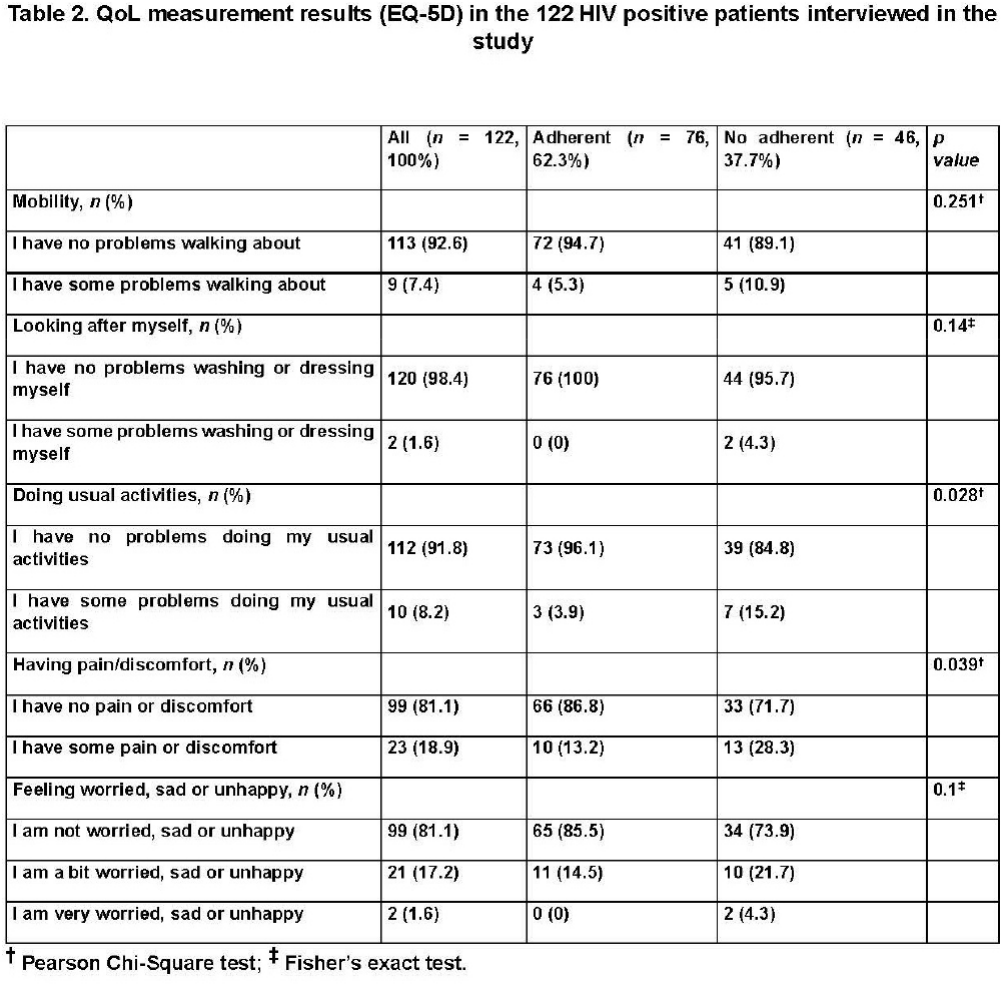

**Methods:**

We conducted an analytical cross-sectional study involving HIV patients on ART for ≥1 year. Participants were recruited from The University Hospital of Caracas-Outpatient Clinic and completed a structured sociodemographic survey and the EQ-5D-3L. Statistical analyses included Kolmogorov–Smirnov, Mann–Whitney U, t-test, chi-square, and Fisher’s exact tests. Binomial logistic regression (backward stepwise Wald selection) identified independent predictors of ART non-adherence. Data were analyzed using SPSS 26, and tables were made with Excel 2019.

**Figure:**
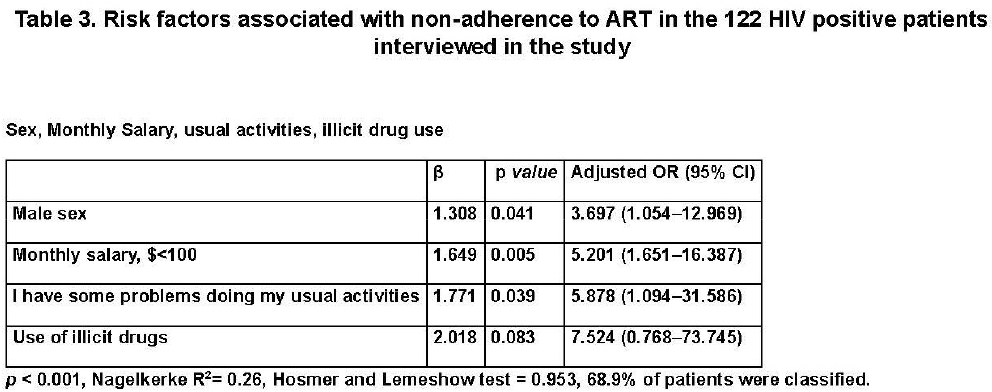

**Results:**

122 patients were interviewed. Key sociodemographic (table 1): male sex (79.5%), intermediate education (54.1%), low-income (73%), recent alcohol use (9.2%), and illicit drug use (4.9%). Most reported no issues in EQ-5D domains (table 2): mobility (92.6%), self-care (98.4%), usual activities (91.8%), pain/discomfort (81.1%), and anxiety/depression (81.1%). Patients were ART-adherent (62.3%) or non-adherent (37.7%). ART non-adherence was significantly associated with extreme poverty (p< 0.001), recent alcohol intoxication (p=0.004), and illicit drug use (p=0.028), as well as reported problems with usual activities (p=0.028) and pain/discomfort (p=0.039) in the EQ-5D scale. Logistic regression (table 3) identified male sex (OR 3.70, 95% CI 1.054–12.969), low-income (OR 5.20, 95% CI 1.651–16.387), and problems with usual activities (OR 5.88, 95% CI 1.094–31.586) as significant risk factors for ART non-adherence.

**Conclusion:**

Assuming confounders are evenly distributed, these findings suggest that socioeconomic hardship and functional limitations are strongly associated with ART non-adherence in this urban Venezuelan population. Interventions targeting social determinants may help improve ART outcomes.

**Disclosures:**

Andrés F. Henao Martínez, MD, MPH, F2: Grant/Research Support|Scynexis: Grant/Research Support

